# Das Hamburger Register für intravitreale Injektionstherapien (QIVOM)

**DOI:** 10.1007/s00347-021-01454-w

**Published:** 2021-08-20

**Authors:** Christian Wolfram, Marc Schargus

**Affiliations:** 1 Universitätsklinikum Hamburg-Eppendorf (UKE), Martinistraße 52, 20246 Hamburg, Deutschland; 2 Asklepios Augenklinik Nord-Heidberg, Hamburg, Deutschland; 3 Klinik für Augenheilkunde, Heinrich-Heine-Universität, Düsseldorf, Deutschland

**Keywords:** Register, Intravitreale Therapie, Anti-VEGF-Antikörper, Patientenorientierung, Deutschland, Register, Intravitreal injections, Anti-VEGF antibodies, Patient orientation, Germany

## Abstract

**Hintergrund:**

Intravitreale operative Medikamenteneingaben (IVOM) gehören zu den häufigsten medizinischen Prozeduren überhaupt mit ca. 1,5 Mio. Eingriffen in Deutschland pro Jahr. Für diese enorme Versorgungsaufgabe gibt es nur wenige empirische Daten über den Versorgungsprozess und seine klinische und subjektive Wirkung.

**Material und Methoden:**

Es wird die Entwicklung und der Aufbau des Hamburger Registers für intravitreale Injektionstherapien (QIVOM) detailliert beschrieben. IVOM-Patienten der drei großen Augenkliniken Hamburgs (Asklepios Kliniken Nord-Hamburg und Barmbek, Universitätsklinikum Hamburg-Eppendorf) werden im Rahmen der Routineversorgung mit IVOM zur Studienteilnahme eingeladen. Es werden subjektive Patientenangaben zum Krankheits- und Behandlungserleben erhoben und diese mit den patientenbezogenen medizinischen Parametern elektronisch ergänzt, pseudonymisiert und in einer elektronischen Datenbank erfasst.

**Ergebnisse:**

Von den ersten 162 Studienpatienten (Alter 41–95 Jahre) war die Behandlungsindikation bei 64% exsudative altersbedingte Makuladegeneration (AMD), bei 22% retinaler Venenverschluss und bei 11% diabetisches Makulaödem. Es ergibt sich ein heterogenes Bild der Erkrankungsschwere und der subjektiven Beeinträchtigung. Eine Sehschärfe von 0,5 und besser wiesen 31,8% der Patienten am behandelten Auge im Vergleich zu 79,1% am Partnerauge auf. Die größte praktische Einschränkung betraf die Lesefähigkeit, bei der über ein Drittel erhebliche Einschränkungen erlebte. Autofahren war immerhin für 62% möglich. Eine Verbesserung der Sehfähigkeit durch IVOM erreichten 45% der Patienten.

**Schlussfolgerung:**

Die Datenerfassung von patientenseitigen Daten und medizinischer Versorgungsdaten aus den Kliniken stellt den großen Vorteil des neu geschaffenen Registers dar. Die Erweiterung der Datenbasis soll in Zukunft vielfältige weiterführende Erkenntnisse erbringen und zur Qualitätssicherung in der IVOM-Therapie beitragen.

## Abstract (en)

**Background:**

Intravitreal drug delivery belongs to the most common medical procedures, with approximately 1.5 million treatments per year in Germany. However, for this enormous challenge to provision of proper care, there is a lack of empirical data regarding the treatment process and its clinical and subjective effect.

**Materials and methods:**

This publication presents the development and structure of the Hamburg register for intravitreal therapies (QIVOM). Patients undergoing intravitreal injection therapy were invited to join the register study. Patients were recruited from the Hamburg eye clinics in Heidberg-Nord and Barmbek as well as from the University Medical Center Hamburg-Eppendorf (UKE). Both subjective patient-reported information about the perception of their eye disease and its treatment as well as medical parameters were stored in a central electronic database.

**Results:**

Of the first 162 study patients (aged 41–95 years), 64% suffered from wet age-related macular degeneration (AMD), 22% had a retinal vein occlusion, and 11% a diabetic macula edema. Disease severity and subjective impairment were heterogenous. Among these patients, 31.8% had a visual acuity above 10/20 on the treated eye compared to 79.1% on the other eye. The reduced ability to read was the most relevant limitation for more than one third of patients. However, 62% were able to drive a vehicle. An improvement of vision through intravitreal therapy was experienced by 45%.

**Conclusion:**

Collection of patient-reported as well as treatment-related data comprises the advantage of the newly created register. This extension of the data basis should deliver new findings in the future and contribute to quality assurance in intravitreal care.

## Hintergrund und Fragestellung

Die Diskrepanz der Ergebnisse klinischer Studien und tatsächlich erreichter klinischer Resultate im Versorgungsalltag hat die Erforschung der Behandlungswirklichkeit („real life“) in den letzten Jahren in den Fokus gerückt. Für verschiedene ophthalmologische Erkrankungen wurden Krankheitsregister aufgebaut, um die Verbreitung und spezifische Versorgung abzubilden [15].

Intravitreale operative Medikamenteneingaben (IVOM) gehören mittlerweile zu den häufigsten medizinischen Eingriffen überhaupt. Für Deutschland berichten Wenzel et al. [22] in ihrer jährlichen Umfrage zur ambulanten Okularchirurgie^1^ über eine Zahl von 497.719 erfassten IVOM-Therapien für 2019, wobei nur 24 % der versendeten Umfragebögen ausgefüllt zurückgeschickt wurden (38 % Rücklaufquote unter ophthalmologischen Hauptabteilungen) und nicht alle Versorgungsstellen erfasst sind, in denen IVOM-Therapien durchgeführt werden. Vorsichtig geschätzt kann man also von einer mindestens dreifachen Häufigkeit der erfassten Zahlen ausgehen, was somit mindestens 1,5 Mio. jährlichen Injektionen für Deutschland entspricht. Trotz dieser enormen Verbreitung als medizinischem Eingriff ist nur relativ wenig über die Versorgungspraxis und die klinische und subjektive Wirkung der Therapien bekannt. So stellt sich etwa der Unterschied zwischen intendierter und erreichter Versorgung besonders dar: Während die großen Zulassungsstudien für Anti-VEGF-Medikamente bis zu 12 Injektionen pro Jahr erzielten [8, 10, 11], wurden in Beobachtungsstudien des Versorgungsalltags nur 4 bis 5 Injektionen pro Jahr erreicht [2, 20, 25]. Weiterhin wurde beschrieben, dass eine höhere Injektionsfrequenz zu besseren Visusergebnissen führte [7, 21]. Um die Diskrepanz zum Versorgungsalltag zu verstehen, ist es notwendig, durch weiterführende Studien (vgl. auch [5]) mehr Informationen über die Perspektive des Patienten, sein Krankheitserleben und sein Verhalten zu erfahren, die Versorgungsabläufe und -routinen näher zu beschreiben und zu hinterfragen, welche klinische und subjektive Wirkung IVOM-Therapien im Alltag tatsächlich erzielen.

Das Hamburger Register für intravitreale Injektionstherapien (QIVOM) soll eine indikationsübergreifende Datenbasis über den Versorgungsalltag bei intravitrealen Injektionen schaffen. Diese dient dem Zweck, sowohl die Strukturen und Prozesse als auch die klinischen und subjektiven Ergebnisse von IVOM-Therapien zu beschreiben und zu analysieren. Dieser Artikel stellt den Aufbau und die Struktur des Registers vor und präsentiert erste Studienergebnisse.

### Studienkonzeption

Die Datenerhebung für das Hamburger Register für intravitreale Injektionstherapien (QIVOM) geht vom Patienten und seiner Erfahrung der zugrunde liegenden Netzhauterkrankung und Versorgungserfahrung aus. Dazu wurde nach einer qualitativen Vorstudie mit zehn Patienteninterviews ein Registerprotokoll entwickelt, das sich an den Vorgaben der Deutschen Netzwerks für Versorgungsforschung [12, 19] orientiert. Es werden subjektive Informationen und Bewertungen durch den Patienten selbst erfasst und ergänzend medizinische Parameter aus der Patientenakte entnommen. Die verschiedenen Parameter sind in Tab. 1 und 2 zusammengefasst.

**Table 1:** 

*Angaben zum Gesundheits- und Krankheitserleben*
– Wahrnehmung der Symptome der Netzhauterkrankung
– Bisheriges Krankheitserleben
– Ein- oder Beidseitigkeit der Erkrankung
– Begleiterkrankungen
– Dauermedikation
– Alltagsfähigkeiten (Autofahren, Lesen, Erkennen von Menschen)
– Erfassung von Gesundheitserleben und Lebensqualität (Fragebogen EQ-5D [4])
– Psychosoziale Versorgung
– Erfassung klinisch symptomatischer Angstzustände (Fragebogen GAD‑2 [24])
– Initiale Therapieerwartungen
– Bewertung der bisherigen Injektionstherapien auf das Sehvermögen
*Erleben der medizinischen Versorgung*
– Erleben des bisherigen Behandlungsverlaufs
– Zeitraum zwischen Diagnosestellung und Erstinjektion
– Häufigkeit von OCT-Kontrollen
– Anzahl bisheriger Injektionstherapien
– Therapieintervall
– Bewertung der Häufigkeit und Schwere von Nebenwirkungen
– Patientenzufriedenheit
– Therapieadhärenz (Unterbrechungen, Abbrucherwägungen)
– Anzahl der Arztbesuche
– Persönlicher Aufwand (Anfahrt, Wartezeit, Begleitung)
*Soziodemografische Angaben*
– Alter
– Geschlecht
– Schul- und Berufsabschluss
– Beruflicher Status
– Familienstand
– Haushaltsstatus
– Pflegebedürftigkeit

**Table 2:** 

– Zentrale Sehschärfe (Dezimalvisus)
– Behandlungsindikation
– Ophthalmologische Komorbiditäten
– Refraktionsstatus
– Linsenstatus
– Augeninnendruck
– Systemische Komorbiditäten
– Bei Diabetikern: Dauer, Blutzuckereinstellung (HbA1c), Insulinpflicht
– Psychologische Komorbiditäten
– Erkrankungsdauer
– OCT-Befund (zentrale Netzhautdicke in Mikrometern am primär behandelten und am Partnerauge)
– Therapieschema
– Therapieverlauf

Insgesamt werden bis zu 69 Fragen vom Patienten beantwortet, dazu gehören neben den Angaben zum Krankheitsverlauf auch neun Fragen zur soziodemografischen Situation. Weiterhin besteht auch die Möglichkeit, offene Kommentare einzufügen. Aus der Patientenakte werden darüber hinaus bis zu 24 weitere klinische Parameter berücksichtigt. Die unterschiedliche Anzahl der Fragen und Parameter ergibt sich, da für besondere Patientengruppen wie Patienten mit Diabetes zusätzliche Items abgefragt werden. Bei den Verlaufsuntersuchungen sind weniger Fragen vom Patienten zu beantworten, und es müssen auch weniger medizinische Informationen aus der Patientenakte extrahiert werden. Im weiteren Verlauf der Registererhebung kann die Datenerhebung modulweise erweitert oder gekürzt werden, wenn sich z. B. besondere neue Fragestellungen ergeben oder falls einzelne Fragen oder Parameter nicht mehr benötigt werden.

Die jeweiligen Fragenkataloge und einzelnen Antwortmöglichkeiten werden als Electronic Case Report Forms (ECRF) für die teilnehmenden Studienzentren auf einer eigenen Webseite (www.qivom.de) veröffentlicht.

### Patienteneinschluss

Es werden derzeit Patienten in die Datenerhebung eingeschlossen, die sich einer intravitrealen Therapie aufgrund einer Netzhauterkrankung unterziehen an einer der Hamburger Augenkliniken Asklepios Nord-Heidberg, Asklepios Barmbek oder dem Universitätsklinikum Eppendorf (UKE). Die Patienten werden in ihrer Wartezeit auf eine Injektionstherapie um die Teilnahme an der Datenerhebung gebeten. Nach Aufklärung und einer schriftlichen Zustimmungserklärung werden die Patienten für etwa 20 min vom Studienpersonal interviewt oder können auch selbst Daten mittels eines Tablet-PC eingeben, sofern es ihr funktionelles Sehvermögen gestattet. Die Patienten müssen über hinreichende Deutschkenntnisse und kognitive Möglichkeiten verfügen, um die jeweiligen Fragen verstehen und beantworten zu können.

### Datendokumentation

Die medizinischen Daten werden anschließend aus der elektronischen Patientenakte (für Asklepios Nord-Heidberg und Asklepios Barmbek FIDUS, Darmstadt, für die Universitäts-Augenklinik Hamburg-Eppendorf ifa systems, Frechen) ergänzt. Die patientenseitigen Angaben und die medizinischen Daten werden in jeweils einem elektronischen Formular (Electronic Case Report Forms – ECRF) pseudonymisiert auf einen zentralen Server, der sich auf dem UKE-Gelände befindet, hochgeladen. Da die Patientendaten bereits bei der Übertragung in die zentrale Datenbank nicht personalisiert sind, ist eine Rückverfolgung zum Einzelnen aus den Registerdaten nicht möglich, sodass ein größtmögliches Level an Datenschutz für die Patienten gewährleistet ist. Der Zugriff auf die Studiendaten ist nur für autorisiertes Studienpersonal möglich. Längerfristig soll eine wissenschaftlicher Beirat eingerichtet werden, der über den Datenzugriff und die jeweilige Datenanalyse entscheidet. Auf diese Weise soll die wissenschaftliche Unabhängigkeit der Registeranalysen gewahrt bleiben.

Die technische Infrastruktur des Registers wurde gemeinsam mit dem Software-Anbieter Swiss4ward (Zürich, Schweiz) entwickelt, der sich auf die Entwicklung von medizinischen Registern und elektronischen Datenbanken spezialisiert hat. Zwei positive Ethikvoten von der zuständigen Ethikkommission der Ärztekammer Hamburg wurden für die bisherigen Studienprojekte eingeholt. (Ethikvoten der Ärztekammer Hamburg: PV7208/PV7202).

Zur Illustration der Registerinhalte stellen wir die Ergebnisse einer ersten explorativen Querschnittsanalyse des bisherigen Studienkollektivs vor. Dazu wurden aus den Angaben durch die Patienten sowie aus den vorliegenden medizinischen Daten bei kategorialen Variablen einfache Häufigkeiten errechnet. Im Fall stetiger Variablen wurden Mittelwerte errechnet.

## Ergebnisse

### Studienkollektiv

In unserer Auswertung lagen subjektive Angaben von 162 Patienten und klinische Informationen aus der Patientenakte von 140 Patienten vor, die sich im Zeitraum zwischen Dezember 2019 und Dezember 2020 in einer der genannten Hamburger Augenkliniken einer IVOM-Therapie unterzogen haben. Das Alter der Studienteilnehmerinnen und -teilnehmer lag zwischen 41 und 95 Jahren (Durchschnitt 75,4 Jahre) bei einer Verteilung nach Geschlechtern von 61 % weiblichen (*n* = 85) und 39 % männlichen Patienten (*n* = 55). Bei 76 % der Patienten erfolgte die Therapie an einem Auge (*n* = 107), bei 24 % (*n* = 33) an beiden Augen. Die Therapieindikation im Patientenkollektiv war in 64 % der Fälle (*n* = 90) eine exsudative AMD, bei 22 % (*n* = 31) ein retinaler Venenverschluss und bei 11 % (*n* = 15) ein diabetisches Makulaödem. Nach Patientenangaben wurde in 65 % der Fälle (*n* = 106) ein vierwöchiges Therapieschema verfolgt, bei 15 % (*n* = 25) ein unregelmäßiges und in 12 % (*n* = 22) ein 6‑ oder mehrwöchiges Schema (ohne Angabe 5 %; *n* = 9). Nach Angaben aus der Patientenakte verfolgten 16 % (*n* = 22) ein initiales monatliches Pro-re-nata(PRN)-Therapieschema, weitere 30 % (*n* = 42) waren im fortgesetzten monatlichen PRN-Schema und sogar 49 % (*n* = 69) im Treat&Extend(TE)-Schema. Bei 5 % (*n* = 7) lag keine Angabe zum Therapieschema vor.

### Verteilung von Visus und zentraler Netzhautdicke

**Figure 1:**
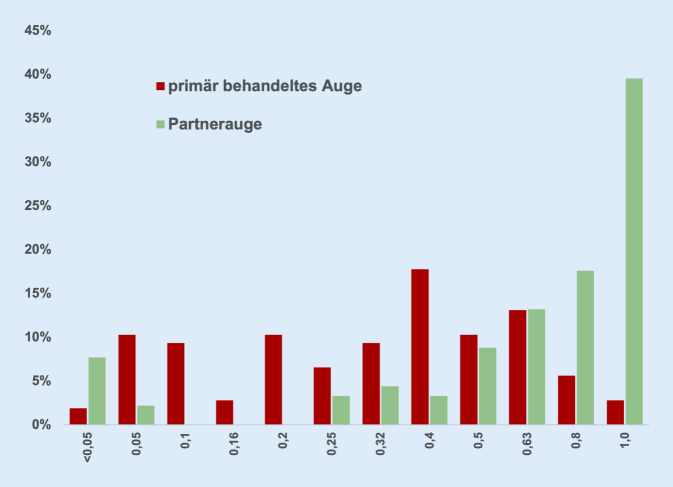

**Figure 2:**
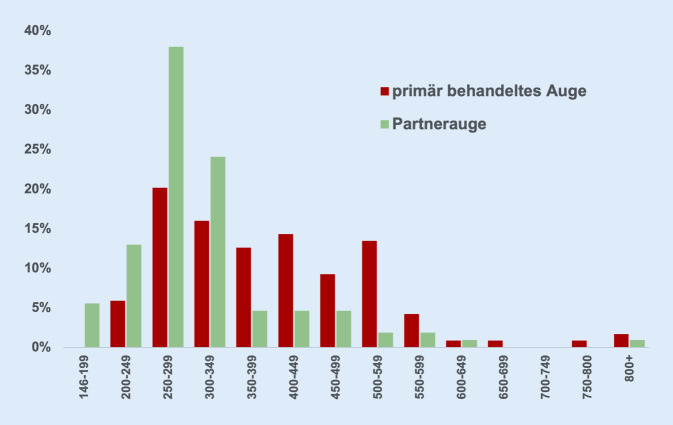

Die Sehschärfe am primär behandelten Auge war in 31,8 % der Fälle 0,5 und besser, am Partnerauge hingegen 79,1 % (Abb. 1). Analog dazu war der Anteil derjenigen Patienten mit einer zentralen Netzhautdicke von weniger als 350 μm am primär behandelten Auge nahezu halb so groß wie am Partnerauge (42 vs. 80 %, Abb. 2).^2^

### Subjektives Erkrankungserleben und funktionelle Einschränkungen

**Figure 3:**
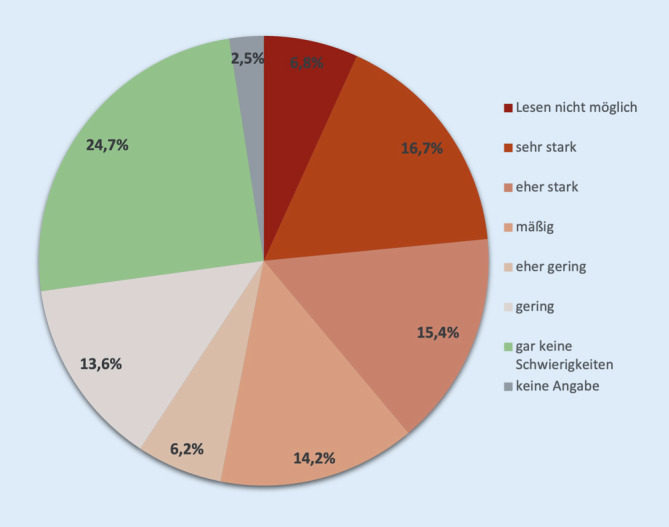

Im Studienkollektiv berichteten 45 % der Patienten von einer schleichenden und 44 % von einer plötzlichen Sehminderung, die zum Behandlungsbedarf für eine IVOM-Therapie geführt habe. 13 % der Patienten gaben an starke Schwierigkeiten beim Erkennen von Menschen zu haben, für 2 % war das sogar unmöglich. Dahingegen hatten weitere 13 % geringe und 59 % gar keine Schwierigkeiten. Lesen dagegen war für 32 % stark eingeschränkt und für 7 % der Patienten sogar unmöglich, wohingegen bei 44 % nur geringe oder gar keine Schwierigkeiten bestanden (Abb. 3).

62 % gaben an, noch Auto fahren zu können, gegenüber 36 %, die dieses nicht taten. Davon nannten 15 %, dass ihr Sehvermögen dafür nicht ausreiche, 12 % führten andere gesundheitliche Gründe an, und 9 % hatten keinen Führerschein. Die lebenspraktischen Beschwerden bestanden für die Betroffenen demnach eher im Lesen als im Erkennen von Menschen oder in der Fahrfähigkeit.

Für die Bewertung der Lebensqualität stellte eine Hälfte der Patienten (50 %) keine oder nur geringe Auswirkungen der Netzhauterkrankung auf die Lebensqualität fest, wohingegen 23 % diese Lebensqualität als „einigermaßen“ und 27 % als stark bis sehr stark beeinträchtigt bewerteten.

### Patientenerwartungen und Therapieergebnisse

**Figure 4:**
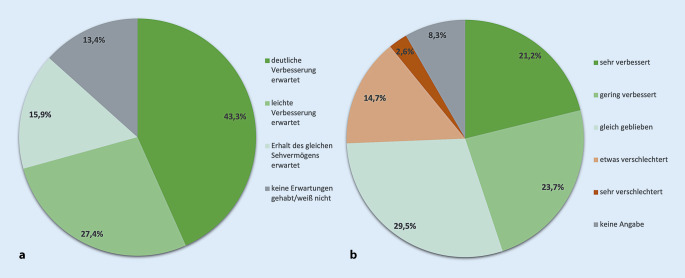

Rückblickend haben zwei Drittel der Befragten bei Therapiebeginn eine Verbesserung durch die Injektionstherapie erhofft. Diese wurde bis zum Zeitpunkt der Untersuchung auch bei 45 % nach eigenen Angaben erreicht, jedoch haben etwa 17 % im Verlauf eine Verschlechterung ihrer Sehfähigkeit erlebt (Abb. 4).

## Diskussion

Das Hamburger Register für intravitreale Injektionen (QIVOM) ermöglicht eine detaillierte Abbildung der Versorgungssituation von Netzhauterkrankungen, die durch intravitreale Injektionstherapien (IVOM) behandelt werden. Konzipiert als längerfristige Registerstudie sollen indikationsübergreifende Erkenntnisse über die IVOM-Versorgung gewonnen und beschrieben werden.

Die ersten explorativen Ergebnisse offenbaren eine große Heterogenität hinsichtlich der Erkrankungsschwere und der Auswirkungen auf die Lebensqualität bei den betroffenen IVOM-Patienten. Während die Mehrheit der behandelten Patienten über einen funktionell relativ guten Visus verfügt und sowohl durch die Therapie als auch durch das Sehen des Partnerauges nur relativ geringe lebenspraktische Einschränkungen erlebt, ergibt sich für etwa ein Drittel der versorgten Patienten ein erheblicher Einfluss auf die subjektive Sehfunktion und Lebensqualität. Naheliegend ist, dass es bei so unterschiedlichen Ausgangspositionen auch deutliche Unterschiede gibt für die Bereitschaft, sich dauerhaft der Therapie zu unterziehen. So wies die AURA-Studie in einer zweijährigen Beobachtungszeit nach, dass ein guter Ausgangsvisus und ein niedrigeres Lebensalter zu Beginn der IVOM-Therapie sowie eine höhere Therapieadhärenz mit einer besseren Visusprognose einhergingen [7]. Longitudinale Untersuchungen des Patientenverhaltens und des Behandlungsverlaufs können die Wechselwirkung zwischen subjektiver Sehfunktion und der Therapieadhärenz weiter ergründen mit dem Ziel, einen vorzeitigen Abbruch von Therapien zu vermeiden [5, 9].

Patienten nehmen die Injektionstherapie sehr unterschiedlich wahr. Bekannt ist, dass viele Patienten Ängste und Schmerzen im Zusammenhang mit der Injektion erleben. In einer qualitativen Untersuchung gaben 56 % der Patienten Ängste an, nach einem klinischen Score für Angst waren 17 % betroffen [17]. In einer anderen Studie lag eine klinisch relevante Angstprävalenz bei 25 % der Patienten vor [16]. Auch für Schmerzen wurden bei 58 % der Patienten in irgendeiner Form Schmerzen bei der Injektion angegeben, wenngleich diese in weit überwiegendem Maß geringfügig und nur für etwa 5 % in klinisch höherem Ausmaß bewertet wurden [14]. Die Beschreibung und Analyse solcher Patientenerfahrungen ist hilfreich, um Hindernisse im Versorgungsweg gezielter verstehen und adressieren zu können. Die Analyse der psychosozialen Begleiterkrankungen, des allgemeinen Gesundheitszustands und der spezifischen Bewertung der Behandlungssituation kann das Verständnis der patientenseitigen Wahrnehmung der IVOM-Therapie vertiefen und ggf. einen besonders vorsichtigen Therapiezugang für sensiblere Patientengruppen anstoßen.

Auffällig ist auch der Unterschied in unseren Ergebnissen zwischen der subjektiven Angabe, nach der sich 65 % der Patienten in einem monatlichen Therapieschema befinden, gegenüber nur 46 %, bei denen gemäß der Patientenakte ein monatliches PRN-Schema verfolgt wird. Anzunehmen ist demnach, dass die Dokumentation eines Treat&Extend-Schemas von Patienten nicht immer als Veränderung des Behandlungsintervalls wahrgenommen wird. Mehr Erkenntnisse über den Wissens- und Informationsstand von Patienten können helfen, mögliche Diskrepanzen zwischen intendierter und erreichter Versorgung zu erklären.

Erfreulicherweise stellen 45 % der Patienten eine subjektive Visusverbesserung unter der IVOM-Therapie fest, wenngleich die Erwartung vor Therapiebeginn nach eigener Auskunft noch höher war. Für die Kontinuität der Therapie über einen längeren Zeitraum ist es wichtig, ob Patienten den Verlauf ihrer Therapie als Erfolg oder als Misserfolg deuten. Die hohe Rate von Nonadhärenz bei der IVOM-Therapie aus anderen Erhebungen – bei AMD bis 32 % und bei Diabetikern sogar bis 44 % [3] – deuten darauf hin, dass es nicht immer ausreichend gelingt, die Patienten von der Therapiefortsetzung zu überzeugen. Therapieabbrüche wurden in der Vergangenheit mit Unzufriedenheit bei etwa einem Drittel oder einer zu intensiven Therapie bei einem Viertel der Betroffenen begründet [6] oder auch mit zu hohen und unrealistischen Therapieerwartungen [18]. Gerade für diejenigen Patienten, die trotz Therapie eine Verschlechterung erfahren – in unserer Untersuchung immerhin 17 % –, stellt sich die Frage der weiteren Therapiemotivation und -fortführung besonders. Mehr Wissen über Diskontinuitäten und Nonadhärenz in der Therapie kann daher dazu beitragen, Versorgungs- und Vertrauensmängel genauer zu identifizieren und die zukünftige Versorgung zu verbessern.

## Ausblick Versorgungsmonitoring

Das hier skizzierte Versorgungsregister für intravitreale Injektionstherapien verfügt über die nötige konzeptionelle und technische Infrastruktur, um auch kontinuierliche Daten über das Versorgungsgeschehen bei IVOM-Therapien zu erheben. Das Ziel ist es, die Datenerfassung über einen längeren Verlaufszeitraum auszudehnen und zu vergrößern, sodass das Register ein wichtiges Instrument zum Monitoring der Versorgung auf mehreren Ebenen werden kann. So ist zunächst auf der Ebene des Patienten die subjektive Bewertung des Krankheitsverlaufs den medizinischen Verlaufsdaten gegenüberzustellen und zu hinterfragen, inwiefern die Bereitschaft der Patienten, sich der IVOM-Therapie zu unterziehen, im längeren Verlauf der Therapie aufrechterhalten bleibt. Auch die Frage, ob sich die Therapieadhärenz je nach Krankheitsschwere, visueller Funktion oder bei beidäugiger Therapie im Kontrast zu einseitiger Behandlung unterschiedlich darstellt, sollte ebenso Gegenstand weiterer Analysen sein wie Fragen nach indikations-, alters- oder geschlechtsspezifischen Unterschieden. Gerade die Erforschung von Therapieabbrüchen bietet die Chance, das Patientenverhalten besser verstehen zu lernen und so Diskontinuitäten noch gezielter entgegenwirken zu können.

Als Instrument der Qualitätssicherung in der IVOM-Therapie ergeben sich aus den Registerdaten auch wichtige Rückschlüsse auf der Ebene der Behandler. So geben Informationen über die Patientenzufriedenheit oder die Wahrnehmung der Abläufe der IVOM-Therapie eine Rückmeldung zur Versorgungspraxis. Konkrete Angaben zur erreichten Injektionsfrequenz, aber auch zur Häufigkeit von schwerwiegenden Nebenwirkungen sind zudem wichtige Parameter der Versorgungsqualität und des Versorgungsmanagements.

Nicht zuletzt auch auf der wissenschaftlichen und gesellschaftlichen Ebene ermöglichen Registerdaten, größere Versorgungsmuster und längerfristige Trends zu beschreiben und zu analysieren (Tab. 3).

**Table 3:** 

– Injektionsfrequenz
– Individuelle Versorgungsverläufe
– Analyse der Therapieadhärenz
– Untersuchung von Therapieabbrüchen und ihren Gründen
– Beschreibung und Erforschung von längerfristigen Versorgungstrends
– Qualitätsberichte für Patienten und Versorger
– Identifikation von Versorgungsunterschieden nach Sektoren/Einrichtungen/Regionen
– Identifikation von unterversorgten Patientengruppen
– Unterstützung klinischer und epidemiologischer Forschung
– Evaluation und Förderung der Patientensicherheit
– Pharmakovigilanzanalysen
– Marktanalysen
– Gesundheitsökonomische Studien
– Unterstützung der Versorgungsplanung

Krankheitsregister haben in der deutschen Augenheilkunde bislang noch keine lange Tradition [15], was umso erstaunlicher ist, da es in Deutschland aufgrund der föderalen Länder- und Sozialversicherungsstrukturen kaum nationale Daten oder valide Routinedatenerhebungen gibt. Staatliche Gesundheitssysteme wie in Schweden können relativ einfach Versorgungsdaten aus einer nationalen Datenbank extrahieren und so etwa die durchschnittliche Injektionsfrequenz (5,9 Injektionen pro Jahr für 2014 für die exsudative AMD) errechnen [23]. Naheliegend wäre es daher, aus medizinischen Routinedaten (z. B. Krankenkassendaten) ähnliche Erkenntnisse für Deutschland ziehen zu wollen. Dieser Zugang erweist sich jedoch als wenig praktikabel, da solche Routinedaten Fehlklassifikationen unterliegen (u. a. durch Überdokumentationen erlösrelevanter Leistungen [13]) und diese zudem keine klinischen Informationen wie z. B. den OCT-Befund oder den Visus abbilden. Ungenaue Klassifikationen, wie z. B. die fehlende Differenzierung der ICD-Codes für exsudative Makulaerkrankungen, die erst im Jahr 2020 geändert wurden, tragen zu dem Problem der zentralen Auswertung zusätzlich bei. Eine höhere externe Validierung und eine detailliertere Beschreibung von Krankheitsverläufen, Komorbiditäten und Behandlungsregimes ermöglicht daher einen Erhebungsansatz aus der Alltagsversorgung, der vom Patienten und seinem Krankheitsleben ausgeht und subjektive und objektive klinische Angaben kombiniert (vgl. [1]).

Der Erkenntnisgewinn der Registerforschung zielt ab auf den Nachweis der Wirksamkeit (Effectiveness) im Versorgungsalltag, die anders ist als Wirkprinzipien und Wirkungen (Efficacy), wie sie randomisierte kontrollierte klinische Studien hervorbringen. Die Antwort einer Versorgungsstudie ist also weniger auf die Frage ausgerichtet, wie ein Therapieverfahren wirkt, als vielmehr darauf, ob und wie dieses im Versorgungsalltag erfolgreich ist. Klinische Studien sind charakterisiert durch eine Limitierung auf selektionierte Patientengruppen und enge Ein- und Ausschlusskriterien. Bei Registerstudien hingegen ist die Zahl der Patienten grundsätzlich nicht limitiert und schließt somit auch heterogene Patientengruppen und weitere Studienzentren ein. Weiterhin werden verschiedene Therapieverfahren und Behandlungskonzepte berücksichtigt, womit idealerweise eine größere Repräsentativität oder externe Validierung erreicht wird [13]. Eine weit höhere Zahl der eingeschlossenen Patienten und der Studienzentren (gerade auch im niedergelassenen Bereich) kann die Aussagekraft der Ergebnisse verbessern, da theoretisch bei einer einseitigen und zu geringen Patientenzahl die Abbildung der Versorgungswirklichkeit auch verzerrt sein kann. Da wir uns in unserer Untersuchung auf stationäre Einrichtungen und die urbane Situation in Hamburg beziehen, ist ein solcher Selektionsbias nicht auszuschließen, wenn es um den Rückschluss auf die Versorgung der Gesamtpopulation geht. Da die untersuchten IVOM-Behandlungen in den Kliniken jedoch alle ambulant durchgeführt wurden, ist anzunehmen, dass die Versorgungsroutinen und Behandlungsregime sich nicht grundlegend unterscheiden vom ambulanten Versorgungssektor. Bei einer größeren Anzahl der untersuchten Patienten könnten dennoch Versorgungsunterschiede zwischen den Versorgungssektoren und auch zwischen einzelnen Einrichtungen ausfindig gemacht werden. Ebenso könnte der Vergleich der Versorgung z. B. mit entlegeneren Landgegenden regionale Versorgungsprobleme genauer identifizieren.

Somit sind die Erkenntnisse aus Registeranalysen in mehrfacher Hinsicht wertvoll: für Versorger im Sinne einer internen Qualitätssicherung und möglicherweise auch eines Benchmarks, für den augenärztlichen Berufsstand, indem die gesundheitsökonomische und gesundheitspolitische Relevanz dieses Versorgungsbereichs verdeutlicht wird, für die Versorgungsplanung durch ein regelmäßiges Monitoring der Versorgungssituation und die Möglichkeit, Versorgungskonzepte zu optimieren, und nicht zuletzt auch für die Patienten selbst für die Wahrung der Patientensicherheit, der Therapieadhärenz und der Zufriedenheit mit der IVOM-Therapie. Eine wirklichkeitsgetreue Abbildung der Versorgungssituation im Register kann somit dazu beitragen, den betroffenen Patientinnen und Patienten realistische Erwartungen für die eigene IVOM-Therapie zu vermitteln.

## Fazit für die Praxis

- Das Hamburger Register für intravitreale Injektionstherapien (QIVOM) soll eine indikationsübergreifende Datenbasis zur Abbildung und Beschreibung der Behandlungswirklichkeit von IVOM-Therapien schaffen.
- Das QIVOM-Register kombiniert subjektive Angaben vom Patienten mit medizinischen Daten aus der Patientenakte.
- Die longitudinale Registererhebung soll zum längerfristigen Monitoring und zur Qualitätssicherung in der IVOM-Therapie beitragen.
- Die erste explorative Querschnittsanalyse zeigt, dass das klinische Bild von Patienten, die sich einer IVOM-Therapie unterziehen, hinsichtlich Visus und OCT-Befund sehr heterogen ist. Auch die persönliche Bewertung des Krankheitserlebens und der Lebensqualität unterscheiden sich erheblich.
- Mehrheitlich gelingt Patienten ein Leben ohne größere Einschränkungen, etwa ein Drittel der Patienten erlebt hingegen schwerere praktische Limitationen.

## Footnotes

1 Beteiligt sind die Augenverbände BDOC, BVA, DGII und DOG.

2 Die Streuungsbreite bei den Angaben der Partneraugen ergibt sich, da hier auch die Fälle beidseitig therapierter Augen berücksichtigt sind.
